# Associations between loneliness and acute hospitalisation outcomes among patients receiving mental healthcare in South London: a retrospective cohort study

**DOI:** 10.1007/s00127-021-02079-9

**Published:** 2021-04-20

**Authors:** Mayur Parmar, Ruimin Ma, Sumudu Attygalle, Christoph Mueller, Brendon Stubbs, Robert Stewart, Gayan Perera

**Affiliations:** 1 Department of Psychological Medicine, Institute of Psychiatry, Psychology and Neuroscience (King’s College London), De Crespigny Park, Box 92, London, SE5 8AF UK; 2 University of London, London, UK; 3 South London and Maudsley NHS Foundation Trust, London, UK

**Keywords:** Loneliness, Mental health, Physical health, Hospitalisation, Emergency admission

## Abstract

**Purpose:**

It is well known that loneliness can worsen physical and mental health outcomes, but there is a dearth of research on the impact of loneliness in populations receiving mental healthcare. This study aimed to investigate cross-sectional correlates of loneliness among such patients and longitudinal risk for acute general hospitalisations.

**Method:**

A retrospective observational study was conducted on the data from patients aged 18 + receiving assessment/care at a large mental healthcare provider in South London. Recorded loneliness status was ascertained among active patients on the index date, 30th Jun 2012. Acute general hospitalisation (emergency/elective) outcomes were obtained until 31st Mar 2018. Length of stay was modelled using Poisson regression models and time-to hospitalisation and time-to mortality were modelled using Cox proportional hazards regression models.

**Results:**

The data from 26,745 patients were analysed. The prevalence of patients with recorded loneliness was 16.4% at the index date. In the fully adjusted model, patients with recorded loneliness had higher hazards of emergency (HR 1.15, 95% CI 1.09–1.22) and elective (1.05, 1.01–1.12) hospitalisation than patients who were not recorded as lonely, and a longer duration of both emergency (IRR 1.06, 95% CI 1.05–1.07) and elective (1.02, 1.01–1.03) general hospitalisations. There was no association between loneliness and mortality. Correlates of loneliness included having an eating disorder (OR 1.67, 95% CI 1.29–2.25) and serious mental illnesses (OR 1.44, 1.29–1.62).

**Conclusion:**

Loneliness in patients receiving mental healthcare is associated with higher use of general hospital services. Increased attention to the physical healthcare of this patient group is therefore warranted.

## Background

Loneliness has been defined as the distress resulting from a perceived discrepancy between an individual’s actual and desired social relationships [1]. It is distinguishable from social isolation as the latter refers to a quantifiable absence of satisfying relationships [2]. The BBC Loneliness Experiment, one of the largest surveys of loneliness in the United Kingdom, reported that the prevalence of loneliness varies within the general population, from 40% in 16–24 year olds to 29% in 65–74 year olds and 27% in adults above 75 years [3]. The total cost of loneliness has been estimated at approximately £11,725 per person over 15 years, including non-medical costs [4]. Loneliness is associated with increased healthcare utilisation in the general population [5] and with worse health outcomes [6, 7]. Amongst a convenience sample of patients in an emergency department, those who scored higher than average on the UCLA loneliness scale used the service 60% more than patients who scored below average [8]. Similarly, a national study in Switzerland reported that loneliness was associated with more visits to a physician as well as worse self-reported physical health throughout the life course [9]. A study of primary care practices has also added to the breadth of evidence, finding that lonely patients had a higher health care utilisation through increased risk of primary care visits, emergency department visits and hospitalisations [10]; however, it has not been established that that loneliness leads to increased physical health service use in all respects [11].

Loneliness is disproportionately prevalent amongst people with mental disorders, observed to be as high as 71% in patients receiving psychiatric rehabilitation in the USA [12]. A study in Australia reported that an estimated 80% of people with a psychotic disorder felt lonely, as compared to 35% of a community control group, adding to existing evidence that serious mental illnesses are associated with loneliness [2, 13]. Depression has been highlighted as a particular risk factor for loneliness, but there is also evidence that a high level of loneliness is associated with worse depressive symptoms [14, 15]. Currently, there is a paucity of evidence comparing physical health outcomes associated with loneliness in patients with mental disorders. One study found that patients with SMI who were lonely were at higher risk of being admitted to mental health inpatient care [16], but acute general hospital admissions have not been evaluated.

Therefore, in a cohort of patients under the care of a secondary mental health service on an index date of 30th Jun 2012, we aimed to investigate factors associated with loneliness, associations of loneliness with acute general hospitalisation and mortality outcomes, and correlates of these outcomes in those identified with loneliness.

## Methods

### Study setting and data source

A retrospective observational study was conducted using the data from the South London and Maudsley NHS Foundation Trust (SLaM) Biomedical Research Centre (BRC) Case Register. SLaM is amongst the largest mental healthcare providers in Europe, serving a catchment area inclusive of four South London boroughs (Lambeth, Lewisham, Southwark and Croydon) with over 1.3 million residents [17]. In 2007–8, the Clinical Record Interactive Search (CRIS) platform was developed with National Institute for Health Research (NIHR) funding. CRIS provides researchers with access to anonymised copies of SLaMs electronic health records within a robust governance framework [18]. Electronic health records comprise structured and unstructured fields of information input routinely by clinicians. The SLaM BRC Case Register has been described in detail [17] and has supported many studies, [19, 20]. The data on over 500,000 cases who had contacts with SLaM since 2006, inclusive of active, inactive or deceased inpatients and outpatients, are currently archived in CRIS [17]. The data from CRIS derived from structured fields in the source electronic health record have been extensively supplemented by the use of natural language processing algorithms to ascertain constructs in free text fields [17]. Likewise, a number of enhancements have been achieved through linkage of CRIS data with those from other sources. The data on acute general hospitalisations within SLaM are derived from a linkage between CRIS and Hospital Episode Statistics (HES) [17]. HES includes statistical abstracts of records of all inpatient, outpatient and emergency care and have been compiled for all healthcare providers in England. The CRIS database and linkages including that with HES have full approval for secondary analysis (Oxford Research Ethics Committee C, reference 18/SC/0372). The study described here was covered by this database approval.

### Sample

The study cohort was selected from SLaM patients who had an ‘active’ SLaM record (i.e., an accepted referral and not discharged from services) on the index date of 30th Jun 2012. Hospitalisation outcomes were measured from the index date until 31st Mar 2018 and mortality was ascertained from the same date until 30th Jun 2020. The exposure, recorded loneliness status, was ascertained within the two years prior to the index date (between 1st Jul 2010 and 30th Jun 2012) and covariates were measured using data within one year before the index date. Patients aged 18 and above at the index date were included in the study.

### Exposure

The primary exposure was loneliness recorded in mental healthcare records, ascertained using a bespoke natural language processing algorithm developed using Generalised Architecture for Text Engineering (GATE) software [21]. In preparation for this project, initial keyword searches were carried out in CRIS to explore terminology used by clinical staff to describe loneliness in the source records. Exploratory search terms were “lonely”, “loneliness”, “perceived social isolation”, “social support”. The terms that were found to commonly refer to the subjective experience of being lonely were used for the subsequent annotation of electronic health records. The terms “lonely” and “loneliness” were found to give the most common description of the experience we aimed to measure. The TextHunter platform [22] was then used to facilitate the annotation of a sample of 1000 random, anonymised SLaM mental healthcare records that included the terms “lonely” and “loneliness”. The annotations determined whether records accurately referred to loneliness–defined by the patient as being or feeling lonely. Annotations also filtered out references to loneliness that did not refer to loneliness, such as loneliness of a family member or forms that contained “lonely” or “loneliness” as a question or heading. Annotations were followed by machine learning algorithm development as it was not feasible to read every patient record in CRIS. The accuracy of the algorithm for identifying recorded loneliness in health records was found to be high (precision = 87%, recall = 100%) on independent checks of 100 records by two annotators. Further details on the development of this application can be found in the SLaM NLP Catalogue [23]. Recorded loneliness was ascertained by the NLP application from health records within two years prior to the index date (30th Jun, 2021). This was a pragmatic decision to cover what was judged to be a sufficient period of time to ascertain recorded loneliness, while avoiding more remote times where loneliness may no longer be relevant to their status on the index date.

### Covariates

Covariates were obtained closest to the index date of 30th Jun 2012 within a maximum of one year preceding this, unless stated otherwise. Sociodemographic information from structured fields included age, gender, ethnicity (Black, White, Asian and Other), cohabiting status and index of multiple deprivation. Cohabiting status grouped being married/having a civil partner and living with someone as ‘cohabiting’ and being divorced, separated, widowed or single were defined as ‘non-cohabiting’, based on the structured marital status codes. The Index of Multiple Deprivation (IMD) is a widely used measure of area-level socioeconomic status derived from seven domains of deprivation (income; employment; health and disability; education, skills and training; barriers to housing and services; crime; and living environment) derived from national Census data [24] and was applied to Lower Super Output Areas (LSOAs) within the SLaM catchment, administrative small area units of 1500–2000 residents. These LSOAs were then ranked by quintile, from least to most deprived [24].

Health of the Nation Outcome Scales (HoNOS) are routinely administered in UK mental health services and recorded as structured data on the electronic health record as a measure of health and social functioning. HoNOS item scores and dates were extracted for the one year up to and including the index date and the closest scores in time were included in analyses. HoNOS items comprise agitation, self-injury, substance use problems, cognitive problems, physical health problems, hallucinations/delusions, depression, relationship problems, daily living problems, living conditions problems and occupational problems [25]. In addition, the presence or not of HoNOS was extracted as a proxy measure of the level and quality of service contact (i.e. as a factor that might influence both the recording of loneliness, if present, and the likelihood of a hospitalisation outcome).

Presence or not of the following psychiatric diagnoses were ascertained from previously recorded ICD-10 codes from structured fields within the electronic health record based on the prior evidence suggesting associations to loneliness: (i) mental and behavioural disorders due to psychoactive substance use [26] (F10*–F19*), (ii) dementia [27] (F00*, F01*, F02*, F03*), (iii) anxiety disorders [28] (F40* to F42*), (iv) eating disorders [29] (F50*), (v) adult personality and behaviour [30] (F60*–F61*), (vi) SMI [2] (F20*, F25* and F31*) and vii) depression [30] (F32* and F33*). Psychotropic medication use was extracted to generate the following covariates: (i) antipsychotics, (ii) antidepressants, (iii) anxiolytics/hypnotics. The use of antidepressant and anxiolytic/hypnotic medication has not previously been investigated in relation to loneliness in cohorts receiving mental healthcare, therefore these factors were considered exploratory in this study. As pain was felt to be a potential correlate of loneliness and thus a confounder for associations with hospitalisation, recorded analgesic medication was also extracted as an exploratory factor [31, 32]. Information on medication use was derived from structured medication fields supplemented by natural language processing algorithms applied to open text fields [23, 33].

Some physical disabilities and diseases have been identified as risk factors or contributory to loneliness. Hearing loss [34], visual impairment [35], osteoporosis [36], Parkinson’s Disease, circulatory diseases [37] as well as higher risk of falling [38] have been specifically highlighted in this respect. Although, urinary tract infections (UTIs) have not been specifically investigated in relation to loneliness, there is evidence of profound social implications of UTIs, such as staying at home and restricted daily functioning [39]. Hence, the presence of UTI was added as an exploratory covariate in our study. With this in mind, reasons for admission to hospital were ascertained within the one year preceding the index date, and the following binary variables were defined on the basis of ICD-10 codes for any recorded discharge diagnosis: hearing loss (H90-95), visual disturbance and blindness (H53-54), osteoporosis (M80-85), Parkinson’s Disease (G20), any circulatory diseases (ICD-10 chapter 1), syncope and collapse (R50-69), UTIs (N39).

### Outcomes

The data were obtained from HES on acute admission to general hospital after the index date and the emergency vs. elective nature of the admission. Emergency admissions were ascertained from ‘admission methods’ codes 21–24 and 28, and elective admissions from codes 11 to 13 in accordance with the NHS data dictionary [40]. The following outcomes were also obtained for elective and emergency hospital admissions separately up until 31st Mar 2018: (i) length of stay (LOS) for the first hospital admission; (ii) number of hospital admissions; (iii) time to the first hospital admission. Finally, time to death was obtained from the index date until a censoring date of 30th Jun 2020, based on a linkage of all SLaM records for past and current patients with the NHS spine for date of death.

### Statistical analysis

Initially, descriptive characteristics of the patients recorded as lonely were when compared with the remainder of patients. Poisson regression analyses (generating incidence rate ratios; IRRs) were carried out to investigate associations of loneliness with number of hospital admissions and length of stay (LOS) as dependent variables for elective/emergency hospital admissions separately. Cox proportional hazards models (generating hazard ratios; HRs) were conducted to investigate associations between loneliness and time-to-event hospitalisation (elective/emergency separately) and death. Finally, survival analyses were carried out for mortality and first emergency hospital admission as an outcome. Models were adjusted for presence of HoNOS, age, gender, ethnicity, cohabiting status, deprivation quintile; HoNOS scores; analgesic and psychotropic medication and psychiatric comorbidity, and in the final model, hospital admissions. Correlates of loneliness were investigated using sociodemographic and health covariates measured on or before index date. Logistic regression models (generating odds ratios; ORs) were used to investigate correlates of loneliness using sociodemographic variables, HoNOS subscales, mental health diagnoses, analgesic and psychotropic medication use and prior specific hospitalisations as independent variables. Finally, a Cox proportional hazard regression model was used to investigate correlates of time-to-event emergency hospitalisation amongst those patients with recorded loneliness.

We hypothesised that patients who were in contact with SLaM and who were recorded as lonely would have increased LOS for the first hospital admission, increased number of hospital admissions and shorter time-to-event emergency hospital admission. There is limited evidence that investigates time-to-event emergency hospitalisation in patients with recorded loneliness; therefore, this analysis was exploratory.

All regression analyses examining correlates (either of loneliness in the full sample; or of health outcomes in the sample restricted to those experiencing loneliness) used backward elimination. This involved starting with all candidate variables from the unadjusted analysis. In the multivariable analysis, factors were eliminated where the *P* value for the association of interest was greater than 0.10, and this process was repeated until no further variables could be deleted without a statistically significant (*P* value < 0.05) loss of it. Akaike’s Information Criteria (AIC) and Bayesian information criteria (BIC) were used to determine the model fit. Furthermore, independent variables that were auto-correlated were eliminated during this process. All statistical analyses were carried out using STATA version 13 (StataCorp, College Station, TX, USA).

## Results

Of the total of 26,745 patients under the care of SLaM on 30th Jun 2012, 4385 (16.4%) had previously been recorded as lonely and were compared with the remaining 22,360. Table 1 displays the characteristics of the cohorts. The mean age was significantly higher for patients recorded as lonely, in whom there was also a greater proportion of older adults (aged 65 +) as compared to the remainder. Those with recorded loneliness were also more likely to be female, in a Black ethnic group, non-cohabiting and living in a more deprived neighbourhood. Problems on all components of the HoNOS were increased in those with recorded loneliness as compared to the comparator group, as were the proportions with all mental health diagnoses investigated apart from mental and behavioural disorders due to psychoactive substance use and dementia. Regarding medications, analgesics, antidepressants and anxiolytics/hypnotics were used more commonly, and antipsychotics less commonly, by those with recorded loneliness as compared to those not recorded as lonely. Patients recorded as lonely had significantly higher likelihood of all types of previous hospitalisation, apart from Parkinson’s disease and visual disturbance/blindness.

**Table 1:** Cohort characteristics of SLaM patients recorded as lonely and not recorded as lonely at index date, 30th June 2012

Characteristics of the population	Recorded as lonely (%)		
	No	Yes	*P* value*
Total population	22,360	4385	
Age			
Mean age (SD)	47.0 (18.4)	48.4 (18.7)	< 0.001
18–39	39.1	36.7	
40–64	43.5	43.5	
65 and over	17.4	19.8	
Gender			< 0.001
Female	47.2	55.9	
Male	52.8	44.1	
Ethnicity			< 0.001
Asian	5.0	5.4	
Black	21.2	27.1	
White	63.9	59.2	
Other	9.4	8.2	
*Missing*	*0.6*	*0.1*	
Cohabiting status			< 0.001
Cohabiting	21.0	12.2	
Non-cohabiting	75.1	86.8	
*Missing*	*3.9*	*1.0*	
Living alone	19.3	32.1	
Index of Multiple Deprivation (IMD)			< 0.001
First quintile (least deprived)	20.7	13.9	
Second quintile	20.2	20.7	
Third quintile	20.1	21.8	
Fourth quintile	19.2	21.5	
Fifth quintile	19.7	22.1	
HoNOS problems^a^			
Agitation problems	9.1	12.8	< 0.001
Self-injury problems	3.1	7.3	< 0.001
Substance use problems	6.5	11.9	< 0.001
Cognitive problems	14.6	18.2	< 0.001
Physical health problems	17.4	27.1	< 0.001
Hallucinations	12.5	20.3	< 0.001
Depressed	19.3	33.2	< 0.001
Relationship problems	18.0	34.0	< 0.001
Daily living problems	19.2	28.3	< 0.001
Living conditions problems	7.5	13.4	< 0.001
Occupational problems	15.8	25.1	< 0.001
* Missing HoNOS data*	*43.3*	*15.4*	
Mental health diagnosis			
SMI	22.7	40.2	< 0.001
Mental and behavioural disorders due to psychoactive substance use	12.2	11.9	0.58
Dementia	7.9	9.0	0.02
Anxiety	4.9	6.5	< 0.001
Personality disorders	2.7	8.5	< 0.001
Eating Disorders	1.5	3.2	< 0.001
Depression	13.2	24.9	< 0.001
Pain relief medication			
Analgesics	16.3	24.9	< 0.001
Psychiatric medication			
Antipsychotics	62.6	40.6	< 0.001
Antidepressants	35.0	56.8	< 0.001
Anxiolytics and hypnotics	26.0	50.3	< 0.001
Previous hospital admission			
Syncope and collapse	8.6	15.3	< 0.001
Hearing problems	0.7	1.1	0.01
Osteoporosis	1.1	1.6	0.01
Visual disturbance and blindness	0.8	1.1	0.05
UTIs	3.5	5.3	< 0.001
Parkinson’s disease	0.5	0.4	0.38
Circulatory diseases	12.4	15.9	< 0.001

^*^Chi-square statistic using degree of freedom is calculated by using the following formula: *DF* (r− 1)(c− 1), where, *DF* degree of freedom, *r* number of rows, *c* = number of columns

^a^Health of the Nation Outcome Scales (HoNOS) is a binary variable with score of 0 and 1 is defined as no problem and score of 2, 3 and 4 is defined as a severe problem

Table 2 describes outcomes for the comparison groups and multivariable models for these associations are displayed in Table 3. After adjustment, patients recorded as lonely were at an increased risk of having a longer LOS during emergency admissions (IRR 1.06, 95% CI 1.05–1.07, *P* < 0.001), greater number of emergency admissions (IRR 1.23, 95% CI 1.19–1.26, *P* < 0.001) and a higher hazard of emergency hospitalisation (HR 1.15, 95% CI 1.09–1.22, *P* < 0.001). Patients recorded as lonely were also at a higher risk of having a longer LOS during elective admissions (IRR 1.02, 95% CI 1.01–1.03, *P*< 0.001), number of elective admissions (IRR 1.17, 95% CI 1.14–1.20, *P* < 0.001) and higher hazard of elective hospitalisation (HR 1.05, 95% CI 1.01–1.12, *P* = 0.04). Mortality hazard did not differ significantly between groups. Notably, adjusting for sociodemographic factors led to decreases in risk ratios for all outcomes relating to emergency hospital admissions, while number of both emergency and elective hospital admissions appeared to be most affected by adjusting for medications and psychiatric comorbidities.

**Table 2:** Outcomes for patients recorded as lonely and not recorded as lonely at index date, 30th Jun, 2012

	Recorded as lonely (%)	
Outcome	No (*n* = 22,360)	Yes (*n* = 4385)
Emergency hospitalisation outcomes		
Had hospital admission	9479 (42.4)	2251 (51.3)
Mean length of stay in days first hospital admission (SD)	16.7 (108.3)	24.1 (132.9)
Mean number of hospital admissions (SD)	1.4(3.3)	2.2 (5.5)
Mean years of follow up until first hospitalisation (SD)	4.0 (2.2)	3.6 (2.3)
Total^a^ LOS in years for first hospital admission since index date	434.1	148.8
Total person years of follow-up	89,945.2	15,568.3
Total LOS in years for 100 person-years of follow-up	0.48	0.96
Elective hospitalisation outcomes		
Had hospital admission	8450 (37.8)	946 (44.4)
Mean length of stay in days first hospital admission (SD)	37.8 (185.0)	63.3 (234.9)
Mean number of hospital admissions (SD)	1.6 (17.4)	2.2 (22.8)
Mean years of follow up until first hospitalisation] (SD)	3.4 (2.2)	3.0 (2.3)
Total LOS in years for first hospital admission since index date	875.3	337.4
Total person years of follow-up	76,665.1	13,331.8
Total LOS in years for 100-person years of follow-up	1.14	2.53
Deceased		
Number deceased	4069 (18.2)	922 (21.0)
Mean years of follow up until death (SD)	7.2 (1.9)	7.1 (2.0)

^a^*LOS*  length of stay

**Table 3:** Regression models for health outcomes comparing patients with recorded loneliness as compared to the remainder of active patients on 30th Jun 2012

Outcome	Unadjusted model^a^	Model 2^b^	Model 3^c^	Model 4^d^	Model 5^e^
First emergency hospital admission					
LOS for first emergency hospital admission (IRR)^f^	1.21 (1.20, 1.23), < 0.001	1.06 (1.05, 1.07), < 0.001	1.05 (1.04, 1.06), < 0.001	1.05 (1.04, 1.06), < 0.001	1.06 (1.05, 1.07), < 0.001
Number of emergency hospital admissions(IRR)^f^	1.53 (1.50, 1.57), < 0.001	1.44 (1.41, 1.48), < 0.001	1.42 (1.38, 1.45), < 0.001	1.27 (1.23, 1.30), < 0.001	1.23 (1.19, 1.26), < 0.001
Time to event emergency hospital admission(HR)^h^	1.25 (1.20, 1.31), < 0.001	1.23 (1.17, 1.29), < 0.001	1.28 (1.22, 1.35), < 0.001	1.17 (1.11, 1.24), < 0.001	1.15 (1.09, 1.22), < 0.001
First elective hospital admission					
LOS for first elective hospital admission(IRR)^f^	1.42 (1.41, 1.43), < 0.001	1.15 (1.14, 1.16), < 0.001	1.13 (1.12, 1.14), < 0.001	1.03 (1.02, 1.04), < 0.001	1.02 (1.01, 1.03), < 0.001
Number of elective hospital admissions(IRR)^f^	1.32 (1.29, 1.35), < 0.001	1.29 (1.26, 1.32), < 0.001	1.33 (1.30, 1.37), < 0.001	1.23 (1.20, 1.26), < 0.001	1.17 (1.14, 1.20), < 0.001
Time to event elective hospital admission(HR)^g^	1.22 (1.16, 1.28), < 0.001	1.16 (1.10, 1.22), < 0.001	1.17 (1.11, 1.24), < 0.001	1.07 (1.01, 1.14), 0.02	1.05 (1.01, 1.12), 0.04
Deceased					
Time to event death(HR)^g^	0.98 (0.92, 1.05), 0.60	1.01 (0.94, 1.09), 0.80	1.05 (0.96, 1.13), 0.28	1.01 (0.93, 1.09), 0.88	1.00 (0.91, 1.08), 0.85

(IRR/HR with 95% confidence interval and *P* values displayed)

^a^Model 1: adjusted for presence of HoNOS data

^b^Model 2: adjusted for age, gender, ethnicity, cohabiting status, deprivation quintile, Living alone

^c^Model 3: Model 2 + HoNOS

^d^Model 4: Model 3 + analgesic and psychiatric medication, psychiatric co-morbidities

^e^Model 5: Model 4 + hospital admissions

^f^Poisson regression model reporting incidence rate ratio (IRR)

^g^Cox proportional hazard model reporting Hazard ratio

Table 4 summarises regression analyses of factors associated with recorded loneliness up to the index date. The results show increasing age and female gender as independently associated with loneliness. Non-cohabiting status was associated with 68% increased odds of recorded loneliness, which was also higher in the fifth quintile of neighbourhood deprivation. Although HoNOS-rated relationship problems were associated with increased odds of being recorded as lonely, no other HoNOS components were. SMI, personality disorders and depression were all associated with being recorded as lonely and an eating disorder diagnosis was associated with the greatest increase in odds of 67%. The four medications investigated in this study were all associated with an increased risk of being recorded as lonely, as was a previous admission to hospital for syncope/collapse.

**Table 4:** Logistic regression for factors associated with recorded loneliness at the index date^a^ (Odds ratio (95% Confidence interval), *P* value)

Covariates	Model 1: unadjusted	Model 2: adjusted for each other	Model 3: adjusted for each other
HoNOS data present	4.19 (3.84, 4.57), < 0.001	^b^	^b^
10-year increase in Age	1.10 (1.05, 1.15), < 0.001	1.04 (1.01, 1.08), 0.01	1.03 (1.01, 1.04), 0.05
Male gender	0.70 (0.66, 0.75), < 0.001	0.80 (0.72, 0.88), < 0.001	0.75 (0.70, 0.82), < 0.001
Ethnicity			
White	Ref	Ref	
Black	1.38 (1.28, 1.49), < 0.001	1.09 (0.97, 1.22), 0.14	
Asian	1.18 (1.02, 1.36), 0.03	0.96 (0.78, 1.18), 0.69	
Other	0.95 (0.84, 1.07), 0.39	1.04 (0.87, 1.23), 0.70	
Non-cohabiting	1.99 (1.81, 2.19), < 0.001	1.69 (1.50, 1.90), < 0.001	1.68 (1.50, 1.89), < 0.001
Living alone	1.98 (1.84, 2.12), < 0.001	1.64 (1.50, 1.80), < 0.001	1.66 (1.52, 1.82), < 0.001
Index of Multiple Deprivation (IMD)			
First quintile (least deprived)	Ref	Ref	Ref
Second quintile	1.52 (1.36, 1.7), < 0.001	1.15 (0.98, 1.36), 0.09	1.13 (0.98, 1.29), 0.08
Third quintile	1.61 (1.45, 1.8), < 0.001	1.23 (1.05, 1.45), 0.01	1.16 (1.01, 1.33), 0.03
Fourth quintile	1.67 (1.49, 1.86), < 0.001	1.23 (1.04, 1.45), 0.01	1.13 (0.99, 1.30), 0.07
Fifth quintile	1.66 (1.49, 1.86), < 0.001	1.30 (1.10, 1.53), < 0.001	1.19 (1.04, 1.36), 0.01
HoNOS problems present^c^			
Agitation problems	0.93 (0.84, 1.03), 0.15		
Self-injury problems	1.59 (1.39, 1.83), < 0.001	1.14 (0.96, 1.37), 0.14	
Drinking problems	1.27 (1.14, 1.41), < 0.001	1.03 (0.91, 1.18), 0.57	
Cognitive problems	0.79 (0.72, 0.86), < 0.001	0.91 (0.80, 1.04), 0.19	
Physical health problems	1.06 (0.98, 1.15), 0.14		
Hallucinations	1.11 (1.02, 1.21), 0.01	0.99 (0.88, 1.12), 0.94	
Depressed	1.25 (1.16, 1.35), < 0.001	1.08 (0.96, 1.20), 0.19	
Relationship problems	1.44 (1.33, 1.55), < 0.001	1.44 (1.30, 1.59), < 0.001	1.42 (1.30, 1.56), < 0.001
Daily living problems	0.98 (0.91, 1.06), 0.69		
Living conditions problems	1.23 (1.11, 1.36), < 0.001	1.01 (0.89, 1.17), 0.78	
Occupational problems	1.09 (1.01, 1.18), 0.04	0.92 (0.82, 1.02), 0.12	
Mental health diagnosis			
SMI	2.29 (2.14, 2.45), < 0.001	1.43 (1.27, 1.61), < 0.001	1.44 (1.29, 1.62), < 0.001
Mental and behavioural disorders due to psychoactive substance use	1.15 (0.96, 1.38), 0.12		
Dementia	1.15 (1.03, 1.29), 0.02	1.05 (0.82, 1.33), 0.70	
Anxiety	1.35 (1.18, 1.55), < 0.001	1.03 (0.84, 1.26), 0.78	
Personality disorders	3.38 (2.96, 3.86), < 0.001	1.59 (1.33, 1.90), < 0.001	1.63 (1.37, 1.94), < 0.001
Eating disorders	2.10 (1.72, 2.57), < 0.001	2.17 (1.65, 2.86), < 0.001	1.67 (1.29, 2.25), 0.03
Depression	2.18 (2.02, 2.36), < 0.001	1.32 (1.17, 1.49), < 0.001	1.35 (1.20, 1.52), < 0.001
Pain relief medication received			
Analgesics	1.70 (1.57, 1.83), < 0.001	1.48 (1.31, 1.68), < 0.001	1.49 (1.32, 1.68), < 0.001
Psychiatric medication received			
Antipsychotics	2.44 (2.28, 2.61), < 0.001	1.17 (1.03, 1.32), 0.01	1.15 (1.02, 1.30), 0.03
Antidepressants	2.44 (2.29, 2.61), < 0.001	1.48 (1.33, 1.64), < 0.001	1.46 (1.32, 1.62), < 0.001
Anxiolytics and hypnotics	2.88 (2.7, 3.08), < 0.001	1.62 (1.46, 1.79), < 0.001	1.63 (1.47, 1.80), < 0.001
Previous hospital admission			
Syncope and collapse	1.93 (1.75, 2.12), < 0.001	1.36 (1.19, 1.59), < 0.001	1.40 (1.22, 1.60), < 0.001
Hearing problems	1.62 (1.17, 2.24), < 0.001	1.03 (0.57, 1.85), 0.93	
Osteoporosis	1.44 (1.10, 1.87), 0.01	0.96 (0.59, 1.56), 0.86	
Visual disturbance and blindness	1.44 (1.05, 1.98), 0.03	1.33 (0.77, 2.31), 0.31	
UTIs	1.53 (1.32, 1.78), < 0.001	1.05 (0.79, 1.39), 0.73	
Parkinson’s disease	0.82 (0.50, 1.33), 0.42		
Circulatory diseases	1.34 (1.22, 1.46), < 0.001	1.11 (0.94, 1.31), 0.21	

^a^Health of the Nation Outcome Scales (HoNOS) is a binary variable with score of 0 and 1 is defined as no problem and score of 2,3 and 4 is defined as a severe problem

^b^Data present/missing were omitted because of collinearity

^c^Model 2: AIC = 15,748.6; BIC = 16,024.8; Model 3: AIC = 15,731.2; BIC = 16,037.4;

Table 5 summarises factors associated with emergency hospitalisation rates in Cox regression models within the subset of patients who were recorded as lonely. Of the sociodemographic variables, both increasing age and being White were associated with higher risk, as were HoNOS-rated physical health and daily living problems, a diagnosis of dementia or disorders due to psychoactive substance use, and analgesic medication use. Increased risk was also associated with previous hospitalisation for syncope/collapse, UTIs or circulatory diseases.

**Table 5:** Cox proportional hazards models of factors associated with emergency hospitalisation among patients recorded as lonely at the index date^a^ (Hazard ratios with 95% CI and *P* values displayed)

Covariates	Model 1: unadjusted	Model 2: adjusted for each other	Model 3: adjusted for each other
10-year increase in age	1.33 (1.22, 1.39), < 0.001	1.56 (1.48, 1.63), < 0.001	1.57 (1.51, 1.63), < 0.001
Male gender	0.83 (0.77, 0.91), < 0.001	1.10 (0.90, 1.34), 0.38	
Ethnicity			
White	Ref	Ref	Ref
Black	0.78 (0.71, 0.86), < 0.001	0.75 (0.59, 0.95), 0.02	0.70 (0.58, 0.84), < 0.001
Asian	0.70 (0.57, 0.85), < 0.001	0.70 (0.42, 1.19), 0.19	0.75 (0.53, 1.06), 0.10
Other	0.65 (0.55, 0.77), < 0.001	0.56 (0.32, 0.98), 0.04	0.47 (0.29, 0.74), < 0.001
Non-cohabiting	0.89 (0.79, 1.04), 0.10		
Living alone	1.08 (0.98, 1.18), 0.12		
Index of Multiple Deprivation (IMD)			
First quintile (least deprived)	Ref		
Second quintile	0.99 (0.86, 1.15), 0.93		
Third quintile	1.00 (0.87, 1.15), 0.99		
Fourth quintile	1.06 (0.92, 1.22), 0.42		
Fifth quintile	1.00 (0.87, 1.15), 0.99		
HoNOS problems present^b^			
Agitation problems	1.25 (1.11, 1.41), < 0.001	1.10 (0.85, 1.43), 0.47	
Self-injury problems	1.06 (0.90, 1.23), 0.50		
Substance use problems	1.16 (1.03, 1.32), 0.02	1.11 (0.81, 1.53), 0.51	
Cognitive problems	1.61 (1.45, 1.78), < 0.001	1.13 (0.88, 1.46), 0.34	
Physical health problems	1.71 (1.56, 1.87), < 0.001	1.42 (1.16, 1.75), < 0.001	1.30 (1.12, 1.51), < 0.001
Hallucinations	1.03 (0.93, 1.14), 0.63		
Depressed	0.84 (0.77, 0.92), < 0.001	0.84 (0.68, 1.05), 0.12	
Relationship problems	0.74 (0.67, 0.81), < 0.001	0.83 (0.67, 1.04), 0.11	
Daily living problems	1.28 (1.17, 1.41), < 0.001	1.60 (1.29, 1.98), < 0.001	1.53 (1.32, 1.77), < 0.001
Living conditions problems	1.10 (0.97, 1.23), 0.13		
Occupational problems	1.11 (1.01, 1.22), 0.03	0.96 (0.77, 1.19), 0.68	
Mental health diagnosis			
SMI	1.04 (0.95, 1.13), 0.40		
Mental and behavioural disorders due to psychoactive substance use	1.29 (1.14, 1.45), < 0.001	1.74 (1.22, 2.47), < 0.001	2.06 (1.61, 2.63), < 0.001
Dementia	2.57 (2.28, 2.89), < 0.001	1.71 (1.26, 2.32), < 0.001	1.51 (1.27, 1.80), < 0.001
Anxiety	0.85 (0.71, 1.04), 0.10		
Personality Disorders	1.26 (1.09, 1.45), < 0.001	1.02 (0.68, 1.52), 0.93	
Eating disorders	0.64 (0.48, 0.84), < 0.001	0.74 (0.30, 1.83), 0.51	
Depression	1.02 (0.93, 1.12), 0.67		
Pain relief medication			
Analgesics	1.75 (1.60, 1.91), < 0.001	1.31 (1.07, 1.62), 0.01	1.25 (1.08, 1.45), < 0.001
Psychiatric medication			
Antipsychotics	1.04 (0.96, 1.13), 0.36		
Antidepressants	1.13 (1.04, 1.23), < 0.001	1.03 (0.84, 1.29), 0.73	
Anxiolytics and hypnotics	1.33 (1.22, 1.44), < 0.001	1.08 (0.87, 1.34), 0.47	
Previous hospital admission			
Syncope and collapse	2.35 (2.13, 2.60), < 0.001	1.27 (1.01, 1.60), 0.04	1.17 (1.01, 1.37), 0.05
Hearing problems	2.79 (2.02, 3.84), < 0.001	1.33 (0.72, 2.45), 0.37	
Osteoporosis	3.51 (2.70, 4.56), < 0.001	0.83 (0.49, 1.39), 0.47	
Visual disturbance and blindness	3.57 (2.64, 4.83), < 0.001	0.59 (0.29, 1.19), 0.14	
UTIs	4.12 (3.56, 4.77), < 0.001	1.37 (1.01, 1.87), 0.04	1.30 (1.07, 1.59), 0.01
Parkinson’s disease	3.30 (2.85, 3.81), < 0.001	1.05 (0.42, 1.66), 0.91	
Circulatory diseases	5.51 (4.83, 6.27), < 0.001	1.30 (1.02, 1.66), 0.04	1.39 (1.18, 1.65), < 0.001

^a^Model 2: AIC = 29,758.6; BIC = 29,932.3; Model 3: AIC = 29,743.6; BC = 29,960.9

^b^Health of the Nation Outcome Scales (HoNOS) is a binary variable with score of 0 and 1 is defined as no problem and score of 2,3 and 4 is defined as a severe problem

## Discussion

Our findings showed a 16.4% prevalence of recorded loneliness in a large cohort of patients receiving mental healthcare from the South London and Maudsley NHS Foundation. Those with recorded loneliness had a higher hazard of hospitalisation, a longer duration of hospitalisation, and a higher number of hospital admissions, applying to both emergency and elective stays and after adjustment for multiple potential confounders. We also found a range of correlates of recorded loneliness in this sample including female gender, non-cohabiting or living alone status, a more deprived neighbourhood of residence, the presence of various mental health conditions including SMI, personality disorders, eating disorders and depression, receipt of analgesics and psychotropic medication including antipsychotics, antidepressants and hypnotics/anxiolytics. Our study found that receiving analgesics, previous hospitalisations due to syncope/collapse, UTIs and circulatory diseases, white ethnic group and use of psychoactive substance were correlated with higher hazard of emergency hospitalisation among patients with recorded loneliness.

Our study provides new evidence on the prevalence of loneliness in a secondary mental health service in the UK with an estimate of 16.4%. In cohorts such as community mental health service users diagnosed with SMI [12] or those in crisis [41], the prevalence of loneliness was reported at 71 and 31% respectively, thus higher than ours in comparison. However, considering other reported international estimates, ours falls within a similar range to that of the general population, reported at around 11–30% for middle age and increasing to 40–50% for older adults [42]. A possible reason for our relatively low prevalence of loneliness within a mental health service is likely to lie in the use of a novel natural language processing application to determine the presence of loneliness – this is considered in more detail under Strengths and Limitations. Most studies use single-item questions, the University of California, Los Angeles (UCLA) Loneliness Scale [43] or the de Jong Gierveld [44] scale which are asked directly from patients, rather than relying on clinician-recorded instances. Speculatively, the stigma associated with loneliness might reduce its recognition within clinical settings or a combination of factors might underlie the low prevalence, such as little medical training dedicated to loneliness as well as patient’s reluctance to raise it at interview [45].

Our study found that patients with recorded loneliness had a higher number of both emergency and elective hospitalisations. Loneliness has previously been associated with greater emergency department use in older adults but not elective hospitalisations [46]. The difference in findings may be a result of our larger dataset that included data of younger and working-age adults as well as post-retirement age groups. We posit that the increase in hospital visits in our cohort could reflect seeking social interactions as a means to mitigate feelings of loneliness. This notion has been supported in the general population and in patients with psychotic disorders [47, 48]. Alternatively, more visits to the hospital could be indicative of decreased buffering: a theorised effect that protects against poorer health through social relationships that provide informational, emotional or tangible resources [49]. Practically, loneliness could lead to poorer medication adherence [50, 51] or possibly lower exposure to social connections that provide health-promoting lifestyle factors, ultimately leading increasing risk of avoidable hospitalisations.

We found that people receiving mental healthcare who were recorded as lonely had longer stays in acute hospitals for medical conditions than those who are not recorded as lonely. Previous evidence has found that both loneliness and mental disorders have separately been linked to longer stays in general hospital [52, 53]. Our findings support the evidence with the combination of both. A longer duration in hospital could have many different explanations. It could be indicative of the severity of the patients’ health, necessitating healthcare over a longer duration. Alternatively, from the perspective of healthcare professionals, an individual who is lonely may not be discharged as quickly because the effects of loneliness on health are known and there may be concerns over the patients’ ability to look after themselves [52]. Further investigation is required to examine the reasons for a longer hospital visit for those who are lonely and receiving mental healthcare for bed management in hospitals.

Our study presents novel findings regarding to time to acute hospitalisation and loneliness for elective and emergency admissions in patients receiving mental healthcare. Those who were recorded as lonely exhibited a higher risk of being admitted to hospital sooner than patients who were not recorded as lonely. Our findings are consistent with those from a previous study where having a smaller or inadequate social network was associated with a higher hazard for emergency hospitalisation in older adults with heart failure [54]. In light of our results, the evidence for the detrimental effects of loneliness on hospitalisation is growing in different cohorts. Loneliness has been linked with poorer health behaviours such as physical inactivity [55] and smoking [56], as well as being a socially stressful experience that could increase risks of immune dysregulation [31]. The combination of any number of these factors could lead to earlier hospitalisation for elective or emergency admissions with patients with mental disorders.

Our study did not find an association between loneliness and mortality within those receiving mental healthcare. Longitudinal evidence has supported a link between loneliness and mortality in the general population, suggesting that loneliness increases the risk of mortality through decrements in emotional and physical health [57]. However, the link is complex and is not always consistently supported. While there are indeed many physical, social and environmental interlinking determinants of loneliness and mortality [49], one study in older adults in Israel found no such link between loneliness and increased mortality [58]. Linking mechanisms suggested by reviewers include alterations to vascular health, obesity, cognitive deterioration and increased inflammatory responses across several age groups which subsequently contribute to mortality [59]. Depression in the presence of loneliness could be contributory to an earlier death perhaps due to motivational depletion, which has the potential to result in negligence towards social aspects of life and taking care of oneself and the associated physical health conditions [60] The effects of loneliness could become more evident over the span of decades due to the cumulative effects, in comparison to years as our study measured, which could explain why there was no association in our study.

There were a range of correlates of loneliness in the cohort. The higher occurrence in females is similar to previously reported findings for older adults [61], and might reflect longer life expectancy increasing the likelihood of being alone for longer or males being less likely to express their loneliness [62]. Our study found that patients who were non-cohabiting were more likely to be recorded as lonely, which is also consistent with research in the general and older adult populations [61]. Furthermore, a recent publication from the English Longitudinal Study of Ageing also found a strong link between area-level deprivation as defined by the IMD and loneliness [63]. In support of existing evidence, mental disorders such as SMI, depression, personality disorders, dementia and eating disorders within our cohort were correlated with loneliness [29, 30]. One paper found that loneliness mediates the relationship between internalised stigma and worsening depressive symptoms as a possible mechanism, which could explain correlation between SMI and loneliness [64]. Similarly, eating disorders have been reported to be associated with negative interpersonal relationships which could have isolating effects on people with this disorder and act as a maintaining factor on the disorder [29, 65]. Few studies have investigated loneliness and personality disorders, although the report by Alasmawi et al. [66] corroborates our finding that personality disorders show strong associations with loneliness, above common mental disorders such as depression and anxiety. Studies have posited, for example, that poorer interpersonal communication and psychosocial factors influence the mechanisms within borderline and schizoid personality disorders [67, 68]. Additionally, we found that patients hospitalised for syncope/collapse were also more likely to be recorded as lonely, which might possibly reflect avoidance of social activity due to fear of falls [69]*.* Falling has been associated with a fear of losing functional independence as well as embarrassment [70]. It is possible therefore that hospitalisation for syncope/collapse may be correlated with loneliness due to similar reasons. While loneliness has been reported to be associated with the development of, and mortality from, circulatory diseases, our study did not concur [71–73]. Given that loneliness has been associated with poorer health behaviours, such as engagement with physical exercise, our finding was thus unexpected [74]. Exploratory analyses in our study found that analgesic medication use was correlated with loneliness. While this has not been directly addressed in prior research, findings from a study on older adults with depression showed that there was increased use of analgesics, due to pain being related to depressive symptomology [75].

We further investigated factors prospectively associated with time-to-emergency hospitalisations among the subset of patients who were recorded as lonely, which as far as we know has not been investigated previously in a mental health cohort. Many associations mirror those found within community samples, such as increased age, physical health problems and daily living problems [76]. An unexpected finding of our study was that patients from a White ethnic group with recorded loneliness were at a higher risk of hospitalisation as compared to those from other ethnic backgrounds. To our knowledge, no studies have investigated the effects of ethnicity and time-to hospitalisation; however, research suggests that White patients are at a lower risk of readmission to hospital within a given amount of time, observed in conditions such as diabetes [77, 78]. Clearly further independent replication and confirmation is required. The association between psychoactive substance use and increased risk of emergency hospitalisation builds on previous research indicating increased likelihood of being readmitted to an emergency department within 12 months of an index visit within this diagnostic group [79], Furthermore, it was has also been suggested that loneliness within an SMI population was associated with poorer self-efficacy in the management of chronic diseases, and that loneliness itself served as a barrier to healthy behaviours [80]. Although our study did not find an association between SMI and a shorter time to hospitalisation, that causal pathway could potentially explain why other disorders were associated with increased hospitalisation risk. Similarly, previous evidence indicates that people with SMI have an increased risk of being hospitalised for urinary conditions, but not for circulatory conditions [81]. Our findings suggested that patients with recorded loneliness are at a higher risk of having an emergency admission if they have previously been hospitalised for UTIs, syncope/collapse and circulatory diseases. While there is some heterogeneity in findings, these do highlight the importance of considering loneliness as a risk factor when conducting analyses for hospitalisation-related outcomes.

### Strengths and limitations

Strengths of this study include the large sample size and near-complete hospitalisation follow-up through the linkage between CRIS and HES databases. In addition, we believe this to be the first study to utilise natural language processing to detect loneliness in routine healthcare, thus allowing its evaluation as an exposure on a large sample. The UCLA loneliness scale [43] and the de Jong Gierveld loneliness scale [44] have received criticism for attempting to quantify a relatively nuanced concept [82], and clearly there may be limitations in the extent to which they can be applied routinely in clinical settings amidst all the other constructs that might be quantified in this way. Clearly the approach taken through natural language processing is to ascertain patients who have specifically said that they feel this way or have been described in that way. This may capture a slightly different construct than a screening scale might, and clearly depends on loneliness being mentioned, identified and recorded. Furthermore, the NLP algorithm itself was focused on lonely or loneliness as recorded entities and did not attempt to capture wider constructs such as social support or contacts. This might, for example account for the absent association with mortality. However, this nonetheless resulted in an exposure that was present in a substantial proportion of the sample and that predicted important health outcomes and; therefore, might be a readily extractable feature in the clinical record around which more ambitious algorithms might be developed.

However, the findings should be considered with the following limitations. First, despite the strengths of natural language processing for determining the cohort, there were some limitations of this. During the timeframe of the study, there was no requirement for clinicians to enter data into patient notes regarding loneliness or status of social support using subject-specific terminology. As a result, the uptake of records by the natural language processing application including the terms “lonely” and “loneliness” may underestimate the number of patients that subjectively felt this way. Second, the cohort only accounted for people within the catchment area who were in contact with the mental health service at the baseline date, who will inevitably represent only a subset of people with given mental disorders. It is conceivable that loneliness is accompanied by reduced access to care, accounting for the lower-than-expected prevalence in the cohort. Third, patients with a confirmed diagnosis may have received more input from services as compared to those who were being assessed or had less severe disorders that did not mandate repeated visits [83]. This could result in more opportunities for patients to report loneliness as compared to those with less severe disorders, as supported by the lower likelihood of a missing HoNOS score (Table 1), which could have resulted in some bias. Fourth, the data within CRIS are administrative in nature and not intended for research purposes. Therefore, many variables previously evaluated as mediating factors between loneliness and health outcomes such as stress, sleep quality and physical activity have not been accounted for [84]. Finally, it would be inappropriate to infer causal relationships underlying the correlates of loneliness due to the cross-sectional nature of this study. It is possible, for example, that loneliness increases likelihood of a mental disorder or vice versa [30]. Longitudinal evidence is required to establish the direction of loneliness and the correlates identified within this study.

### Implications

Despite these limitations, this study contributes new findings on loneliness in patients receiving mental healthcare. Given a recent publication reviewing the costs surrounding loneliness [4] and the findings from this study, there is justification for pursuing interventions to tackle loneliness beyond older adults, with the inclusion of all age groups. The review concludes that a range of interventions are cost-effective for reducing rates of loneliness [4] which could ultimately reduce the risk and number of acute general hospital admissions. A review of existing interventions found that changing social cognition was promising, although more robust evidence was needed [82]. There is discussion within this field regarding the lack of success of interventions, primarily due to the highly idiosyncratic nature of loneliness [85]. Thus far, the majority of interventions have taken a “one size fits all” approach, potentially explaining why they have not been as useful as expected. To address the evident health outcomes of loneliness, person-centred care may be a positive step forward.

A loneliness screening tool has been recommended by the Office of National Statistics for surveys to standardise research [86] and clinical practice may benefit from incorporating more routine screening for loneliness so that it may be identified and addressed sooner. In common with Holt-Lunstad et al. [6], our study demonstrates the negative health outcomes associated with loneliness, additionally indicating that this is a prominent issue for the mental health population and their increased acute general hospital usage. Future research within secondary mental health settings may capitalise on such a routine screening of loneliness to develop research in this area.

## Conclusion

In conclusion, our study contributes important findings to the field of loneliness, specifically that patients who are receiving mental healthcare are at an increased risk of spending a longer time within acute general hospital admissions, having more admissions and be hospitalised sooner than patients who are not lonely. In order to benefit individual patients and reduce the time spent in hospitals, it may be beneficial to investigate interventions that target loneliness for people with mental disorders.
